# Integrative Proteo-Genomic Analysis for Recurrent Survival Prognosis in Colon Adenocarcinoma

**DOI:** 10.3389/fonc.2022.871568

**Published:** 2022-06-30

**Authors:** FeiYan Ai, Wenhao Wang, Shaojun Liu, Decai Zhang, Zhenyu Yang, Fen Liu

**Affiliations:** ^1^ Department of Gastroenterology, The Third Xiangya Hospital of Central South University, Changsha, China; ^2^ Hunan Key Laboratory of Non-Resolving Inflammation and Cancer The Third Xiangya Hospital, Central South University, Changsha, China; ^3^ Department of Breast and Thyroid Surgery, The First Affiliated Hospital of University of South China, Hengyang, China

**Keywords:** colon adenocarcinoma, recurrence, prognosis, proteo-genomics, survival (MeSH)

## Abstract

**Background:**

The survival prognosis is the hallmark of cancer progression. Here, we aimed to develop a recurrence-related gene signature to predict the prognosis of colon adenocarcinoma (COAD).

**Methods:**

The proteomic data from the Clinical Proteomic Tumor Analysis Consortium (CPTAC) and genomic data from the cancer genomic maps [The Cancer Genome Atlas (TCGA)] dataset were analyzed to identify co-differentially expressed genes (cDEGs) between recurrence samples and non-recurrence samples in COAD using limma package. Functional enrichment analysis, including Gene Ontology (GO) and Kyoto Encyclopedia of Genes and Genomes (KEGG) pathway was conducted. Univariate and multivariate Cox regressions were applied to identify the independent prognostic feature cDEGs and establish the signature whose performance was evaluated by Kaplan–Meier curve, receiver operating characteristic (ROC), Harrell’s concordance index (C-index), and calibration curve. The area under the receiver operating characteristic (ROC) curve (AUROC) and a nomogram were calculated to assess the predictive accuracy. GSE17538 and GSE39582 were used for external validation. Quantitative real-time PCR and Western blot analysis were carried out to validate our findings.

**Results:**

We identified 86 cDEGs in recurrence samples compared with non-recurrence samples. These genes were primarily enriched in the regulation of carbon metabolic process, fructose and mannose metabolism, and extracellular exosome. Then, an eight-gene-based signature (CA12, HBB, NCF1, KBTBD11, MMAA, DMBT1, AHNAK2, and FBLN2) was developed to separate patients into high- and low-risk groups. Patients in the low-risk group had significantly better prognosis than those in the high-risk group. Four prognostic clinical features, including pathological M, N, T, and RS model status, were screened for building the nomogram survival model. The PCR and Western blot analysis results suggested that CA12 and AHNAK2 were significantly upregulated, while MMAA and DMBT1 were downregulated in the tumor sample compared with adjacent tissues, and in non-recurrent samples compared with non-recurrent samples in COAD.

**Conclusion:**

These identified recurrence-related gene signatures might provide an effective prognostic predictor and promising therapeutic targets for COAD patients.

## Introduction

Colon adenocarcinoma (COAD) as the primary pathological type of colon cancer is one of the most commonly diagnosed malignancies and also a major cause of cancerous death throughout the world (1, 2). It was reported that there was an increasing approximately 1.1 million and 0.5 million deaths in COAD per year all over the world (3, 4). So far, continuous advancement of surgical technology has improved the survival rate of patients with COAD (5). However, the prognosis of patients, especially at advanced stage, remains poor due to high recurrent rate (6–8). Consequently, it is necessary to identify more characteristic and valuable biomarkers for the early prediction and treatment of COAD recurrence.

The evidence to date suggests the critical roles for abnormal expression of messenger RNAs (mRNAs) in COAD tumorigenesis by regulating a variety of biological processes, including cell proliferation, migration, and invasion. For example, FOXA1 expression was significantly higher in COAD tissues and associated with worse prognosis, which promoted cell proliferation, migration, and invasion in COAD cells (9). TBX19 mRNA expression was significantly increased in tumorous tissues compared to that in non-tumorous tissues, and increased TBX19 mRNA expression was associated with positive lymph node metastasis on colorectal carcinogenesis (10). Similarly, TMPRSS13 silencing in colorectal cancer cell lines increased apoptosis and impaired invasive potential (11). Moreover, bioinformatics analysis based on the public databases are believed to provide valuable information in disease prediction with the rapid development of gene sequencing technology (12, 13). The Cancer Genome Atlas (TCGA) database (14) and Gene Expression Omnibus (GEO) (15) have publicized various mRNA sequence data, which may provide novel information to improve the understanding of the molecular mechanisms of action in the tumorigenesis and progression of COAD. On the other hand, The Clinical Proteomic Tumor Analysis Consortium (CPTAC) consortium is aimed at characterizing the protein inventory in tumors by leveraging the latest developments in mass spectrometry-based discovery proteomics (16). Nowadays, integrating mRNA data and protein data thus helps to more exactly establish a genetic marker and prognostic model that can predict the recurrent survival prognosis of COAD patients.

The current study aimed to integrate the COAD genome and proteome expression level data to screen the factors that were significantly related to the recurrence of COAD at both mRNA and protein expression levels. We further screened the genes that were significantly related to the recurrent prognosis of COAD by combining the clinical prognosis information of the sample and constructed an efficient prediction model of recurrence prognosis by combining clinical factors.

## Materials and Methods

### Data Acquisition

The RNAseq data consisted of 372 COAD samples with a complete set of clinical information, including 78 recurrent and 294 non-recurrent samples downloaded from The Cancer Genome Atlas (TCGA, https://portal.gdc.cancer.gov/) uploaded up to the August 15, 2020. Meanwhile, the protein expression profiles consisted of 34 COAD samples with corresponding clinical information, including 6 recurrent and 28 non-recurrent samples obtained from the Clinical Proteomic Tumor Analysis Consortium (CPTAC, https://cptac-data-portal.georgetown.edu/) database. In addition, we downloaded the two gene expression datasets (GSE17538 and GSE39582) of COAD from GEO database based on GPL570 Affymetrix Human Genome U133 Plus 2.0 Array according to the following criteria: (a) colon adenocarcinoma, (b) the organism is *Homo sapiens*, (c) samples containing recurrent information, and (d) COAD sample size exceeding 150 samples.

### Identification of Co-Differentially Expression Genes

The limma package of R3.6.1 Version 3.34.7 (17) was used to identify the differentially expression genes (DEGs) between recurrent and non-recurrent samples in TCGA COAD samples. Similarly, the differentially expressed proteins (DEPs) were identified between recurrent and non-recurrent samples in CPTAC database using the limma package. A false discovery rate (FDR) <0.05 and |log_2_ fold change (FC)| >0.263 were set as the criteria value for DEGs and DEPs. Visual hierarchical cluster analysis was conducted to display the volcano plot of DEGs and DEPs. The intersection of the DEGs and DEPs was visualized *via* Venn diagrams, which was considered the set of significant co-differentially expressed genes (cDEGs) for further analysis.

### Function Enrichment Analysis

To reveal the functions of cDEGs, the Database for Annotation, Visualization and Integrated Discovery (DAVID, http://david.ncifcrf.gov/) version 6.8 (18, 19) was used to perform Gene Ontology (GO) and Kyoto Encyclopedia of Genes and Genomes (KEGG) pathway analysis. A *p* < 0.05 was considered to be statistically significant.

### Screening of Independent Prognostic cDEGs

First, cDEGs significantly associated with prognosis for overall survival (OS) were screened by performing univariate Cox regression analysis with the survival R3.6.1 package (version R2.41.1; http://bioconductor.org/packages/survivalr/) (20). Afterwards, the multivariate cox regression analysis was carried out to extract independent prognostic cDEGs for OS using survival package in R 3.4.1. For univariate and multivariate Cox regression analyses, the log-rank *p* < 0.05 was thought of as the cutoff for the significant correlation.

### Establishing the Prognostic Model and Performance Evaluation

We then established a prognostic score model of independent prognostic cDEGs for OS according to the following formula: prognostic score (PS) = ∑β_cDEGs_ × Exp_cDEGs_. Here, the β_cDEGs_ represented the estimated contribution coefficient of independent prognostic cDEGs in the multivariate Cox regression analysis, and Exp_cDEGs_ denoted the level of independent prognostic cDEGs. According to this formula, the PS of each sample was calculated in training dataset and two validation datasets (GSE17538 and GSE39582). Next, all patients in each dataset were divided into high- and low-risk groups with the median of the PS as the cutoff criterion. The Kaplan–Meier survival analysis was used to analyze the survival difference between two risk groups. The corresponding *p*-value was calculated with the R3.6.1 survival package (20). The predictive performance of the gene panel for OS was estimated using a time-dependent ROC curve by the “survivalROC” package in R software (21).

### Screening of Independent Prognostic Parameters

The independent clinical prognostic parameters were identified by performing univariate and multivariate Cox regression analyses using the survival package in R 3.4.1 with log-rank *p* < 0.05 as the threshold for significance (20). Following this, Kaplan–Meier curves were used to further explore the relationships between independent prognostic factors and survival prognosis.

### Building and Validating a Predictive Nomogram

Based on all independent prognostic parameters, we built a composite nomogram in the R package “rms” (https://cran.r-project.org/web/packages/rms/index.html) (22, 23) to predict the probability of 3- and 5-year OS for COAD. The calibration curve of the nomogram was plotted by calibrating function of R software to compare predicted OS against observed OS. The predicted and observed outcomes of the nomogram was assessed by using the concordance index (C-index) with a bootstrap method. The area under the ROC curve (AUC) was calculated to make a comparison for discriminatory ability of above prognostic parameters. Subsequently, we compared the nomogram including all with those including only one independent prognostic factor using the area under the ROC curve (AUROC), C-index, and log-rank *p*-value. The same methods were used in the validation dataset 1 to validate the results.

### Tissue Sample Preparation

Fresh tumor tissues and adjacent non-tumor colon tissues (at least 5 cm away from tumor edge) were collected from 60 cases of COAD patients who underwent surgery at the Third Xiangya Hospital of Central South University for mRNA detection. The patients were enrolled with signed written informed consent according to the following inclusion criteria (1): no neoadjuvant chemotherapy or radiotherapy and (2) no other history of surgery. The study was performed in accordance with the ethical guidelines of the Declaration of Helsinki and approved by the Ethics Committee of the Third Xiangya Hospital of Central South University.

### Quantitative Real-Time PCR Analysis

Total RNA was extracted from tissue samples using TRIzol reagent (Invitrogen, Carlsbad, CA, USA), and reverse transcription was performed using PrimeScript™ RT reagent kit (TaKaRa Bio, Inc., Otsu, Japan). Next, quantitative real-time PCR was carried out using Premix Ex Taq™ II (TaKaRa Bio, Inc.) with the following primer sequences, namely, ADH4 (forward, 5′-GGCTGCAATCAACAATGCCA-3′; reverse, 5′-CCAGGGCTTTAGCCTTCACA-3′), AHNAK2 (forward, 5′-CGCGATGTGCGACTGC-3′; reverse, 5′-TCCGTGAGTCCCCTGAATCT-3′), MMAA (forward, 5′-CTTCCGTGGCTTCGGGC-3′; reverse, 5′-AAGCCATCTGACAGCAGCAT-3′), DMBT1 (forward, 5′-TGTCAGCACAGTGAAGACGC-3′; reverse, 5′-TACTGTCGATGCAGGCAAGG-3′), and GAPDH (forward, 5′-GGTGAAGGTCGGAGTCAACG-3′; reverse, 5′-GCATCGCCCCACTTGATTTT-3′) according to the reaction conditions: 95°C for 1 min, then 40 cycles of 95°C for 10 s, 60°C for 30 s, and 75°C for 30 s. Relative mRNA expression levels were calculated with the 2^−ΔΔCq^ method.

### Western Blot Analysis

Total protein sample was extracted from tissue specimens using radioimmunoprecipitation assay (RIPA) buffer in the presence of protease and phosphatase inhibitors (Thermo Fisher Scientific, Waltham, MA, USA Inc.). After quantified with a bicinchoninic acid (BCA) protein assay, 30 μm of total protein were separated on 12% sodium dodecyl sulfate–polyacrylamide gel electrophoresis (SDS-PAGE) and then transferred onto polyvinylidene fluoride (PVDF) membranes (Bio-Rad, Hercules, CA, USA). The membranes were blocked with 5% skim milk dissolved in phosphate-buffered saline (PBS) and Tween 20 (PBST) for 2 h at room temperature, followed by incubated with primary antibodies against CA12 (15180-1-AP, Proteintech Group, Inc., Chicago, Il, USA), AHNAK2 (ab164994, Abcam), MMAA (ab264418, Abcam), DMBT1 (ab276422), and GAPDH (10491-1-AP, Proteintech Group, Inc., Chicago, Il, USA) at 4°C overnight. After incubating with horseradish peroxidase-conjugated secondary antibodies at room temperature for 2 h, protein signals were detected by SuperSignal West Pico Chemiluminescent Substrate (Thermo Fisher Scientific, Waltham, MA, USA Inc.) with GAPDH as the internal control.

### Statistical Analysis

Statistical analyses and graphical presentations were conducted by GraphPad Prism Software 6.0. Quantitative variables were analyzed using a t-test for paired samples or a non-parametric Wilcoxon rank-sum test for unpaired samples as appropriate. Data were expressed as mean ± SD of three independent repeats. The statistical significance was established at *p* < 0.05.

## Results

### Differential Expression Analysis

**Figure 1:**
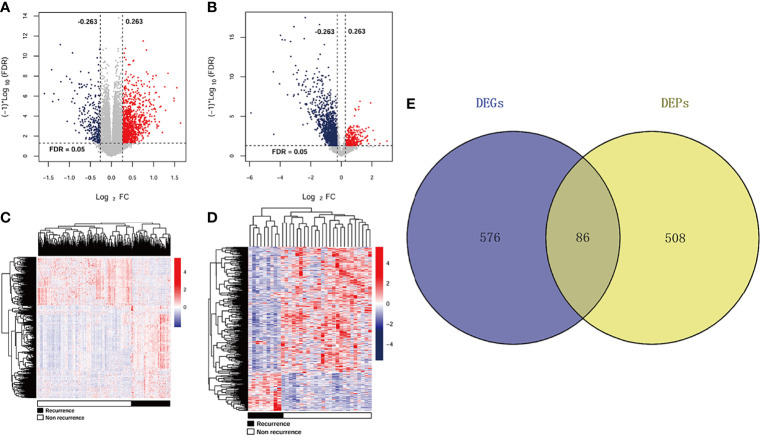
DEGs and DEPs expression profiles in TCGA and CPTAC datasets. A volcano plots of 662 DEGs **(A)** and 594 DEPs **(B)**. Blue and red rots represented significantly downregulated and downregulated genes or proteins, respectively. Dotted lines represented FDR < 0.05, and vertical dashed lines represented |log_2_ FC| > 0.263. Heat maps of 662 DEGs **(C)** and 594 DEPs **(D)** with FDR < 0.05 and |log_2_ FC| > 0.263. Red: higher expression; blue: lower expression. Black and white represented recurrent and non-recurrent samples, respectively. **(E)** Venn diagram shows the intersecting cDEGs from TCGA and CPTAC. Blue area: TCGA dataset; yellow area: CPTAC dataset; cross area: DEGs expressed in both databases.

A total of 662 DEGs (163 downregulated and 499 upregulated) and 594 DEPs (509 downregulated and 85 upregulated) were identified between recurrent and non-recurrent samples based on the threshold of FDR < 0.05 and |log_2_ FC| > 0.263. The expression changes of 662 DEGs (**Figure 1A**) and 594 DEPs (**Figure 1B**) were displayed by volcanic maps. Subsequently, the expression values of 662 DEGs (**Figure 1C**) and 594 DEPs (**Figure 1D**) were hierarchically clustered, and the results were presented in the form of heat maps. These maps clearly distinguished the recurrent samples from non-recurrent samples. The Venn package was used to screen the cDEGs from both databases and generate the Venn map (**Figure 1E**). Finally, 86 recurrent-related cDEGs with high reliability were obtained (**Supplementary Table S1**).

### Functional Annotation for cDEGs

**Figure 2:**
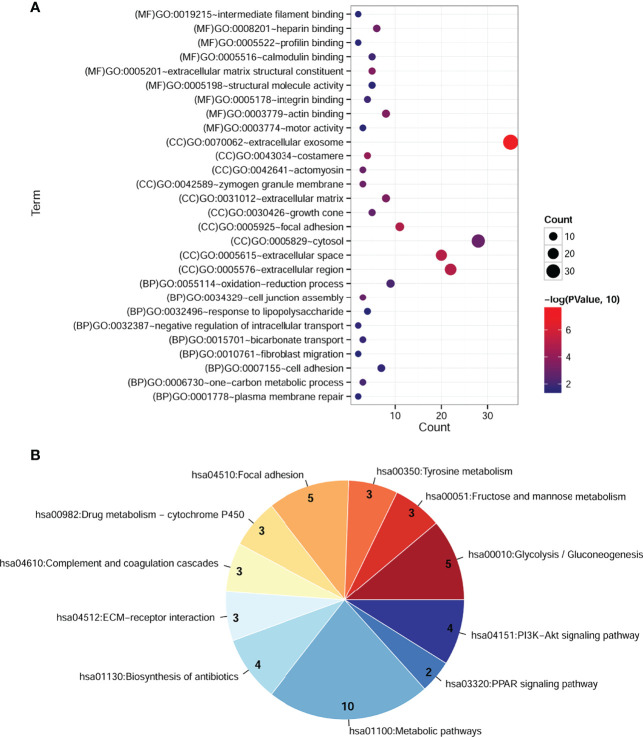
Significantly enriched GO terms and KEGG pathways of cDEGs in COAD. **(A)** Enriched GO terms in the “biological process,” “cell composition,” and “molecular function” category. Different colors indicate different significances, while different sizes indicate the number of genes. **(B)** Enriched KEGG pathways. The color from blue to red indicates the change in significant *p-*value from small to large, and the number represents the gene counts involved in the corresponding pathway.

To explore the potential function of cDEGs, online software DAVID was used for the GO and KEGG pathway analysis. As shown in **Figure 2A**, GO annotations of cDEGs were divided into biological process (BP), cell composition (CC), and molecular function (MF). A total of 35 terms (10 gene counts per term), including 9 BP, 17 CC, and 9 MF were arranged in ascending order according to *p*-value. After screening, it was found that these cDEGs were mainly enriched in oxidation–reduction process and carbon metabolism. In addition, these cDEGs were enriched in 11 KEGG pathways, including metabolic pathway, biosynthesis of antibiotics, and PI3K–Akt signaling pathway (**Figure 2B**).

### Screening of Independent Prognostic Feature cDEGs

The univariate Cox regression analysis was performed to identify prognostic cDEGs, and the results revealed that 42 cDEGs were significantly correlated with recurrent survival prognosis. After further screening, the results from multivariate cox regression analysis (**Table 1**) showed that a total of eight cDEGs were found to be independently related to recurrent survival prognosis, including carbonic anhydrase 12 (CA12), hemoglobin subunit beta (HBB), neutrophil cytosolic factor 1 (NCF1), kelch repeat and BTB domain containing 11 (KBTBD11), metabolism of cobalamin associated A (MMAA), deleted in malignant brain tumors 1 (DMBT1), AHNAK nucleoprotein 2 (AHNAK2), and fibulin 2 (FBLN2).

**Table 1:** The list of independent prognostic feature cDEGs.

Symbol	Coef	P value	Hazard ratio	95%CI
CA12	0.44816	4.360E-04	1.565	1.220-2.010
HBB	0.21914	1.451E-02	1.245	1.044-1.484
NCF1	1.15846	1.722E-02	3.185	1.228-8.262
KBTBD11	-0.55455	1.933E-02	0.574	0.361-0.914
MMAA	-1.04285	3.953E-02	0.352	0.131-0.951
DMBT1	-0.13052	4.151E-02	0.878	0.774-0.995
AHNAK2	0.40594	4.541E-02	1.501	1.300-2.268
FBLN2	-0.37102	4.927E-02	0.690	0.448-0.963

Coef, Coefficient derived from multivariate cox regression analysis; 95%CI, 95% confidence interval.

### Establishing the Prognostic Model and Performance Evaluation

Subsequently, the expression levels of these eight independent prognostic cDEGs in training dataset and two validation datasets were calculated, and prognostic model was constructed as follows:


$$\begin{array}{l} PS=\left(0.44816\right)\times Exp_{CA12}+\left(0.21914\right)\times Exp_{HBB}+\left(1.15846\right)\times Exp_{NCF1}+\\ \left(-0.55455\right)\times Exp_{KBTBD11}+\left(-1.04285\right)\times Exp_{MMAA}+\left(-0.13052\right)\times Exp_{DMBT1}+\\ \left(0.40594\right)\times Exp_{AHNAK2}+\left(-0.37102\right)\times ExpFBLN2 \end{array}$$

**Figure 3:**
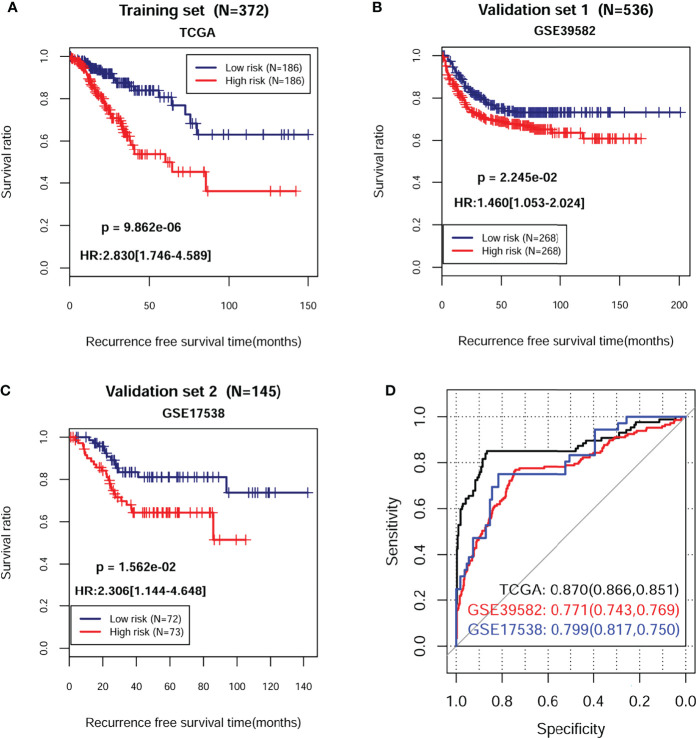
Kaplan–Meier survival and time-dependent ROC analysis in training and validation sets. The KM curve based on PS and survival outcomes in training dataset **(A)**, validation dataset 1 **(B)**, and validation dataset 2 **(C)**, respectively. The red and blue lines, respectively, represent high- and low-risk samples. **(D)** Time-dependent ROC analysis of the eight independent prognostic cDEGs in training dataset, validation dataset 1, and validation dataset 2. HR, hazard ratio; ROC, receiver operating characteristic.

The PS for each sample in each dataset was then calculated accordingly. Based on the median value of PS, all samples in each dataset were classified into high-risk group and low-risk group. The survival analysis revealed that there was a significant correlation between two different risk groups and survival outcomes in training dataset (**Figure 3A**; HR = 2.830; 95% CI, 1.746–4.589; log-rank *p* = 9.862e−06), validation dataset 1 (**Figure 3B**; HR = 1.460; 95% CI, 1.053–2.024; log-rank *p* = 2.245e−02), and validation dataset 2 (**Figure 3C**; HR = 2.306; 95% CI, 1.144–4.648; log-rank *p* = 1.562e−02). ROC curve showed that the AUC for OS was 0.870 in the training dataset, 0.771 in the validation dataset 1, and 0.799 in the validation dataset 2, indicating a better predictive performance in established prognostic model (**Figure 3D**).

### Screening of Independent Prognostic Parameters

**Figure 4:**
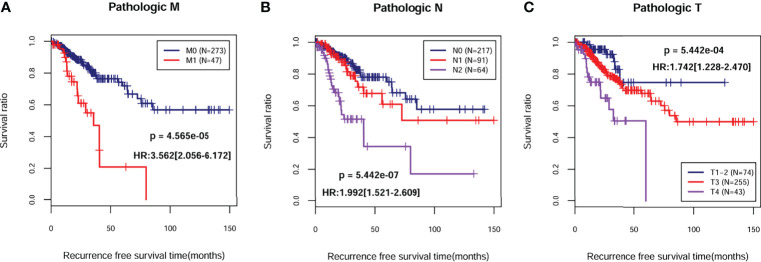
Kaplan–Meier survival analysis of independent prognostic parameters. Kaplan–Meier curve comparing the survival rate between different degrees of pathologic M **(A)**, N **(B)**, and T **(C)** in TCGA-COAD cohort.

**Figure 5:**
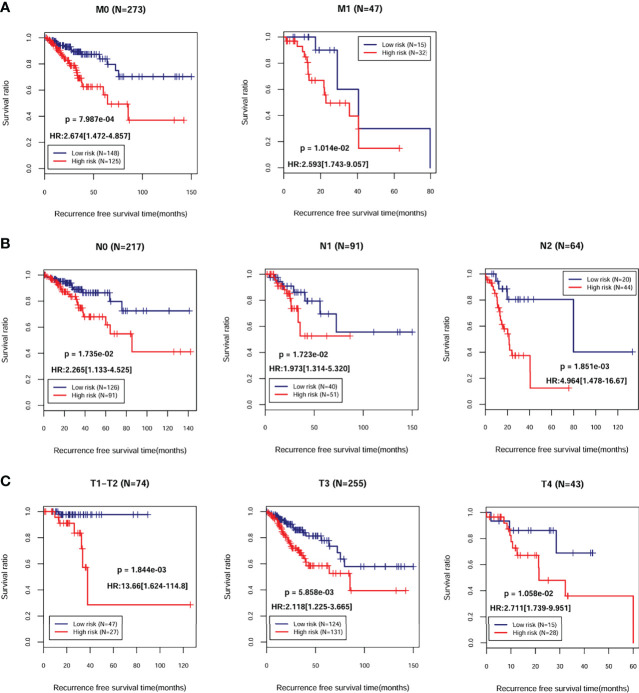
Kaplan–Meier survival analysis of the eight-gene signature in independent prognostic parameters. Kaplan–Meier curve comparing the survival rate between high- and low-risk groups in patients in different pathologic M **(A)**, N **(B)**, and T **(C)**.

A total of 372 patients from TCGA-COAD database with complete information including gender and clinical stage were included for further analysis. Univariate and multivariate Cox regression analyses indicated that pathological M, N, T, and PS model status calculated from the eight-gene signature were independent prognostic parameters for OS (**Table 2**). Moreover, survival analysis showed that pathological M (**Figure 4A**; HR = 3.562; 95% CI, 2.056–6.172; log-rank *p* = 4.565e−05), N (**Figure 4B**; HR = 1.992; 95% CI, 1.521–2.609; log-rank *p* = 5.442e−07), and T (**Figure 4C**; HR = 1.742; 95% CI, 1.228–2.470; log-rank *p* = 5.442e−04) were markedly related to overall survival in COAD patients from TCGA database. Furthermore, we divided the samples according to different pathological M, N, and T and analyzed the association between high- and low-risk group in the samples of each stage. The results indicated that the recurrent-free survival prognosis of the low-risk group was significantly better than that of the high-risk group in patients from pathological M (**Figure 5A**), N (**Figure 5B**), and T (**Figure 5C**) regardless of their stage.

**Table 2:** The univariable and multi-variable cox regression of clinical parameters and survival outcomes of patients with COAD.

Clinical characteristics	TCGA (N=372)	Uni-variable cox		Multi-variable cox	
		HR (95% CI)	P value	HR (95% CI)	P value
Age (years, mean ± SD)	65.95±12.70	0.989 [0.972-1.007]	2.30E-01	–	–
Gender (Male/Female)	201/171	1.594 [1.007-2.523]	4.37E-02	1.225 [0.744-2.019]	4.25E-01
Pathologic M (M0/M1/-)	273/47/52	3.562 [2.056-6.172]	4.57E-05	3.313 [1.155-9.503]	2.59E-02
Pathologic N (N0/N1/N2)	217/91/64	1.992 [1.521-2.609]	5.44E-07	1.634 [1.160-2.781]	7.03E-03
Pathologic T (T1/T2/T3/T4)	10/64/255/43	2.227 [1.431-3.467]	2.62E-04	1.730 [1.524-3.141]	7.19E-03
Pathologic stage (I/II/III/IV/-)	61/145/109/47/10	1.811 [1.394-2.353]	6.23E-06	1.052 [0.309-1.371]	2.59E-01
KRAS mutation (Yes/No/-)	18/25/329	0.657 [0.238-1.812]	4.11E-01	–	–
Microsatellite instability (Yes/No/-)	10/73/289	0.586 [0.135-2.543]	4.44E-01	–	–
Colon polyps present (Yes/No/-)	75/133/164	0.939 [0.528-1.672]	8.31E-01	–	–
Colon polyps history (Yes/No/-)	99/209/64	0.885 [0.503-1.559]	6.71E-01	–	–
Radiation therapy (Yes/No/-)	10/321/41	0.741 [0.669-6.888]	1.88E-01	–	–
PS model status (High/Low)	186/186	2.830 [1.746-4.589]	9.86E-06	2.508 [1.472-4.276]	7.27E-04
Recurrence (Yes/No)	78/294	–	–	–	–
Recurrence free survival time (months, mean ± SD)	29.61±25.63	–	–	–	–

SD, Standard deviation; HR, Hazard ratio; 95%CI, 95% confidence interval; TCGA, The Cancer Genome Atlas.

### Building and Validating a Predictive Nomogram

**Figure 6:**
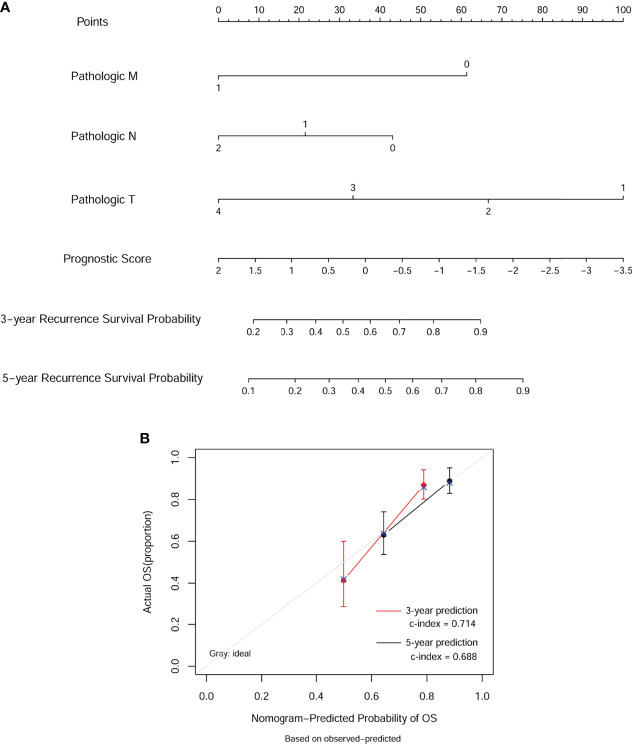
Nomogram predicting overall survival for COAD patients. **(A)** For each patient, three lines are drawn upward to determine the points received from the four predictors in the nomogram. Then, a line is drawn downward to determine the possibility of 3- and 5-year overall survival of COAD. **(B)** The calibration plot for internal validation of the nomogram. The Y-axis represents actual survival, and the X-axis represents nomogram-predicted survival.

Next, we built a nomogram to predict 3- and 5-year OS in the 372 COAD patients using four independent prognostic parameters, including pathological M, N, T, and PS. As shown in **Figure 6A**, the score of every indicator was presented by points scale located at the top of nomogram. After summing the points of each indicator, we could estimate the survival probability at 3 and 5 years. Moreover, we plotted a calibration curve to evaluate the performance of the nomogram model. As described in **Figure 6B**, the C-index was 0.714 and 0.688 for predicted probability of OS at the 3 and 5 years, respectively, suggesting that the constructed nomogram prediction for survival rates showed good agreement with the actual observation for OS patients. Furthermore, compared with nomogram including only the pathological M, N, T, clinical, and PS model, the combined model (**Table 3**) showed the largest AUROC, C-index, and smallest *p*-value for 3-year survival in both training dataset (**Supplementary Figure 1SA**) and validation dataset 1 (**Supplementary Figure 1SB**). Taking together, these results indicated that the nomogram built with the combined model might be the best nomogram for predicting short-term survival, in comparison with nomograms built with a single prognostic factor.

**Table 3:** Comparison of the nomogram with different models.

Type	AUROC (95% CI)		C-index		P Value	
	TCGA	GSE39582	TCGA	GSE39582	TCGA	GSE39582
**Pathologic M**	0.556 (0.908,0.402)	0.505 (0.847,0.463)	0.580	0.516	5.592E-03	1.770E-01
**Pathologic N**	0.593 (0.622,0.564)	0.584 (0.861,0.506)	0.617	0.604	2.937E-03	3.336E-02
**Pathologic T**	0.558 (0.425,0.869)	0.515 (0.825,0.523)	0.587	0.560	5.357E-03	1.439E-02
**Clinical model**	0.744 (0.730,0.585)	0.579 (0.769,0.583)	0.645	0.610	1.762E-04	1.388E-02
**Prognostic score model**	0.870 (0.878,0.833)	0.771 (0.746,0.769)	0.683	0.611	2.85E-06	3.14E-02
**Combine model**	0.951 (0.929,0.939)	0.777 (0.797,0.715)	0.714	0.666	3.71E-08	3.45E-04

AUROC, area under the ROC curve; CI, confidence interval; C-index, concordance index.

### Validation of mRNA by Quantitative Real-Time PCR

**Figure 7:**
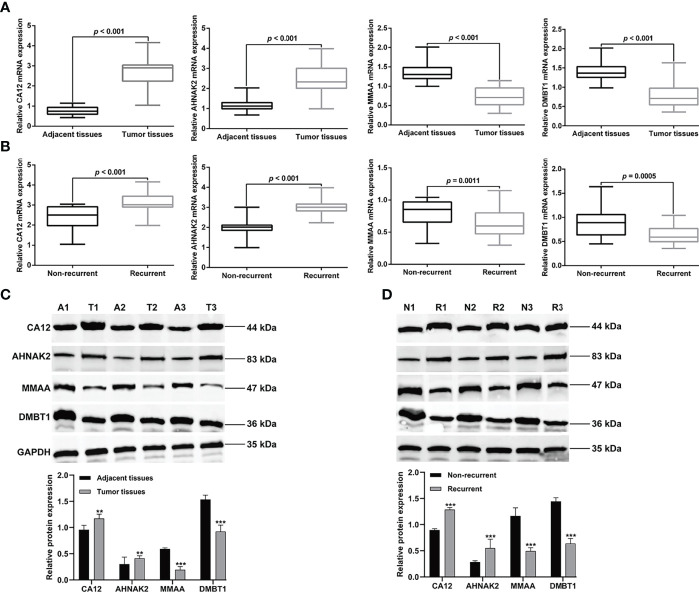
Validation of eight independent prognostic cDEGs. Quantitative real-time PCR analysis was performed to determine the mRNA expression levels of ADH4, AHNAK2, MMAA, and DMBT1 in tumor tissue and adjacent tissues **(A)**, and in recurrent tissues and non-recurrent tissues **(B)**. Western blot analysis was utilized to measure the protein levels of ADH4, AHNAK2, MMAA, and DMBT1 in tumor tissue and adjacent tissues **(C)**, and in recurrent tissues and non-recurrent tissues **(D)**. Data were expressed as mean ± SD of three independent repeats. **p < 0.01, ***p < 0.001, compared with adjacent or non-recurrent tissues. A, adjacent tissues; T, tumor tissues; N, non-recurrent; R, recurrent.

According to the results analyzed by bioinformatics, the mRNA expression levels of eight independent prognostic feature cDEGs selected was then validated in clinical tissues originating from COAD patients. Consistent with the results obtained with bioinformatics, it was revealed that CA12 and AHNAK2 mRNA levels were significantly upregulated in tumor tissues compared to adjacent tissues, whereas MMAA and DMBT1 mRNA levels were downregulated analogously (**Figure 7A**). Moreover, we divided tissue samples into recurrent and non-recurrent samples and further analyzed the mRNA expression levels of the above four genes. As expected, CA12 and AHNAK2 mRNA levels were significantly upregulated in recurrent tissues compared to non-recurrent tissues, whereas MMAA and DMBT1 mRNA levels were downregulated analogously (**Figure 7B**). Consistently, Western blot analysis obtained the same protein trends between tumor tissues and adjacent tissues (**Figure 7C**) and between recurrent and non-recurrent samples (**Figure 7D**).

## Discussion

COAD remains one of the most life-threatening malignancies in the world due to the complex molecular mechanisms. With the development of gene sequencing technology, some potential gene markers, including INHBA (24), COL8A1 (25), and GABRD (26), correlated with poor prognosis for COAD patients have been identified. Nevertheless, the number of such biomarkers in COAD tumorigenesis is still limited. Therefore, it is urgently needed to screen out more biomarkers with higher prediction accuracy for improving the prognosis and outlining an individualized treatment plan for COAD patients.

In this study, by combined TCGA and CPTAC database, a total of 86 cDEGs between recurrent and non-recurrent samples were identified. After univariate and multivariate Cox regression analysis, we finally obtained eight-related risk signatures (CA12, HBB, NCF1, KBTBD11, MMAA, DMBT1, AHNAK2, and FBLN2) that were related to recurrent OS. KM survival analysis and AUC further illustrated that our model had a great predictive performance. Additionally, the prognostic scores of the recurrence-free survival model could be considered as independent predictive indicators. Finally, the nomogram and calibration chart showed that the risk signatures could accurately assess the survival of COAD patients. Carbonic anhydrase 12 (CA12) is a transmembrane protein, which was found to be differentially expressed in colon cancer (27) and a useful clinical tool in discriminating the prognosis of cervical cancer (28). Hemoglobin subunit beta (HBB) is a member of the globin family and a structurally conserved group of proteins often containing the heme group, which has been identified as a potential serum biomarker for the diagnosis and prognosis of ovarian cancer (29, 30). Neutrophil cytoplasmic factor 1 (NCF1) belongs to the NADPH oxidase complex and is a cytoplasmic component, which plays a role in tumor growth and metastasis (31). The study by Kelkka et al. (32) demonstrated that mice lacking NCF1 exhibited reduced growth of implanted melanoma and carcinoma tumors. Kelch repeat and BTB domain containing 11 (KBTBD11) is a member of the KBTBD subfamily of proteins that possess a BTB domain and Kelch repeats, which has been identified as a negative regulator of osteoclast differentiation (33) and reported to promote adipocyte homeostasis (34). In addition, Li et al. (35) manifested the association of high fibulin 2 gene (FBLN2) expression with worse disease-specific survival and metastasis-free survival in urothelial carcinoma.

Next, we selected four genes (CA12, MMAA, DMBT1, and AHNAK2) from the above eight risk biomarkers for quantitative PCR (qPCR) and Western blot analysis verification according to literature retrieval. The experimental results confirmed that ADH4 and AHNAK2 were significantly upregulated, while MMAA and DMBT1 were downregulated analogously in tumor tissues or recurrent tissues compared with controls. Consistent with our data, CA12 is highly expressed in glial tumors compared with normal tissue and predicts for poor clinical course of tumor patients (36). However, Watson et al. (37) put forward an opposite conclusion; they believed that CA12 expression was associated with a better prognosis in an unselected series of invasive breast carcinoma patients. Metabolism of cobalamin-associated A (MMAA) gene is essential for the proper functioning of a cofactor of the methylmalonyl-CoA mutase, whose mutation was associated with the clinical treatment effects of methylmalonic aciduria (38). Malignant brain tumor 1 (DMBT1) was decreased in cervical squamous cell carcinoma (CSCC), whereas its overexpression cannot only inhibit the proliferation, migration, and invasion but also induce the apoptosis of human CSCC cells (39), which strongly supported our results. AHNAK nucleoprotein 2 (AHNAK2), also known as C14orf78, is a member of the AHNAK family, which also includes its homologous gene AHNAK (40). Upregulated ANHAK2 activates the PI3K/AKT signaling pathway and promotes melanoma cell metastasis (41). In agreement with our data, AHNAK2 was upregulated in tumor samples and correlated with poor prognosis in lung adenocarcinoma patients (42). Recent studies also revealed that ANHAK2 could act as a biomarker for various tumors, including thyroid cancer (43), pancreatic ductal cancer (44), bladder cancer (45), and gastric cancer (46). However, the unrecognized roles of CA12, MMAA, DMBT1, and AHNAK2 in COAD are worth further investigating to identify their biological functions and underlying mechanisms in the development and progression of the disease.

Subsequently, KM curve, ROC curve, and risk plot analyses verified that the eight-based risk signature performs well in stratifying the risk groups of recurrent COAD in TCGA. Our data also indicated that the nomogram built with the combined model might be the best nomogram for predicting short-term survival, in comparison with nomograms built with a single prognostic factor.

## Conclusions

In summary, our results suggest that the eight-gene-based recurrent prognosis model is a reliable tool in risk stratification and serves as an independent prognostic factor of the OS in recurrent COAD patients. These model genes are expected to be diagnostic and treatment indicators, which will face the challenge on how to apply various genes signature reasonably in a particular stage of COAD.

## Data Availability Statement

The original contributions presented in the study are included in the article/**Supplementary Material**, further inquiries can be directed to the corresponding author.

## Ethics Statement

The study was performed in accordance with the ethical guidelines of the Declaration of Helsinki and approved by the Ethics Committee of the Third Xiangya Hospital of Central South University. The patients/participants provided their written informed consent to participate in this study.

## Author Contributions

FL made substantial contributions to conception and design of the research. FA and WW carried out data collection and analysis. SL and DZ were involved in drawing figures and tables. DZ and ZY wrote the paper. FA, SL, and ZY edited the manuscript and provided critical comments. All authors contributed to the article and approved the submitted version.

## Funding

This work was supported by The New Xiangya Talent Projects of the Third Xiangya Hospital of Central South University (No. JY201601).

## Conflict of Interest

The authors declare that the research was conducted in the absence of any commercial or financial relationships that could be construed as a potential conflict of interest.

## Publisher’s Note

All claims expressed in this article are solely those of the authors and do not necessarily represent those of their affiliated organizations, or those of the publisher, the editors and the reviewers. Any product that may be evaluated in this article, or claim that may be made by its manufacturer, is not guaranteed or endorsed by the publisher.

## Supplementary Material

Supplementary Figure 1 Performance of gene-based nomogram in predicting survival probability. Time-dependent ROC curves of the pathologic M, N, T, clinical model and combined model in OS prediction in the training dataset **(A)** and validation dataset 1 **(B)**. OS, overall survival; ROC, receiver operating characteristic.
