# Supplementary figures and images for: Integrative Proteo-Genomic Analysis for Recurrent Survival Prognosis in Colon Adenocarcinoma
Source: Front Oncol. 2022 Jun 30;12:871568. doi: 10.3389/fonc.2022.871568 (PMC9281446; doi:10.3389/fonc.2022.871568)

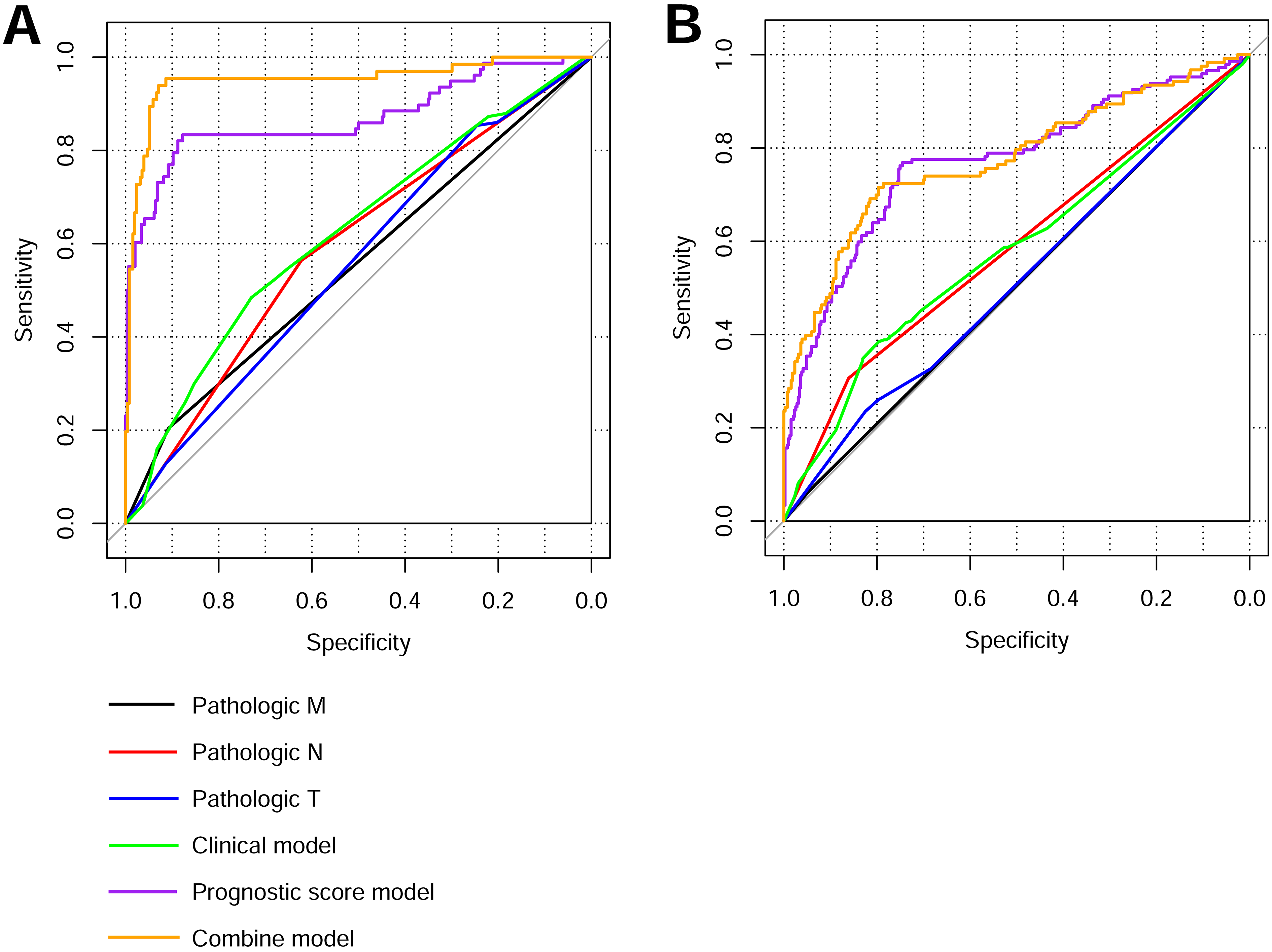

Supplement: Supplementary Figure 1 — Performance of gene-based nomogram in predicting survival probability. Time-dependent ROC curves of the pathologic M, N, T, clinical model and combined model in OS prediction in the training dataset (A) and validation dataset 1 (B). OS, overall survival; ROC, receiver operating characteristic. [file Image_1.tiff]

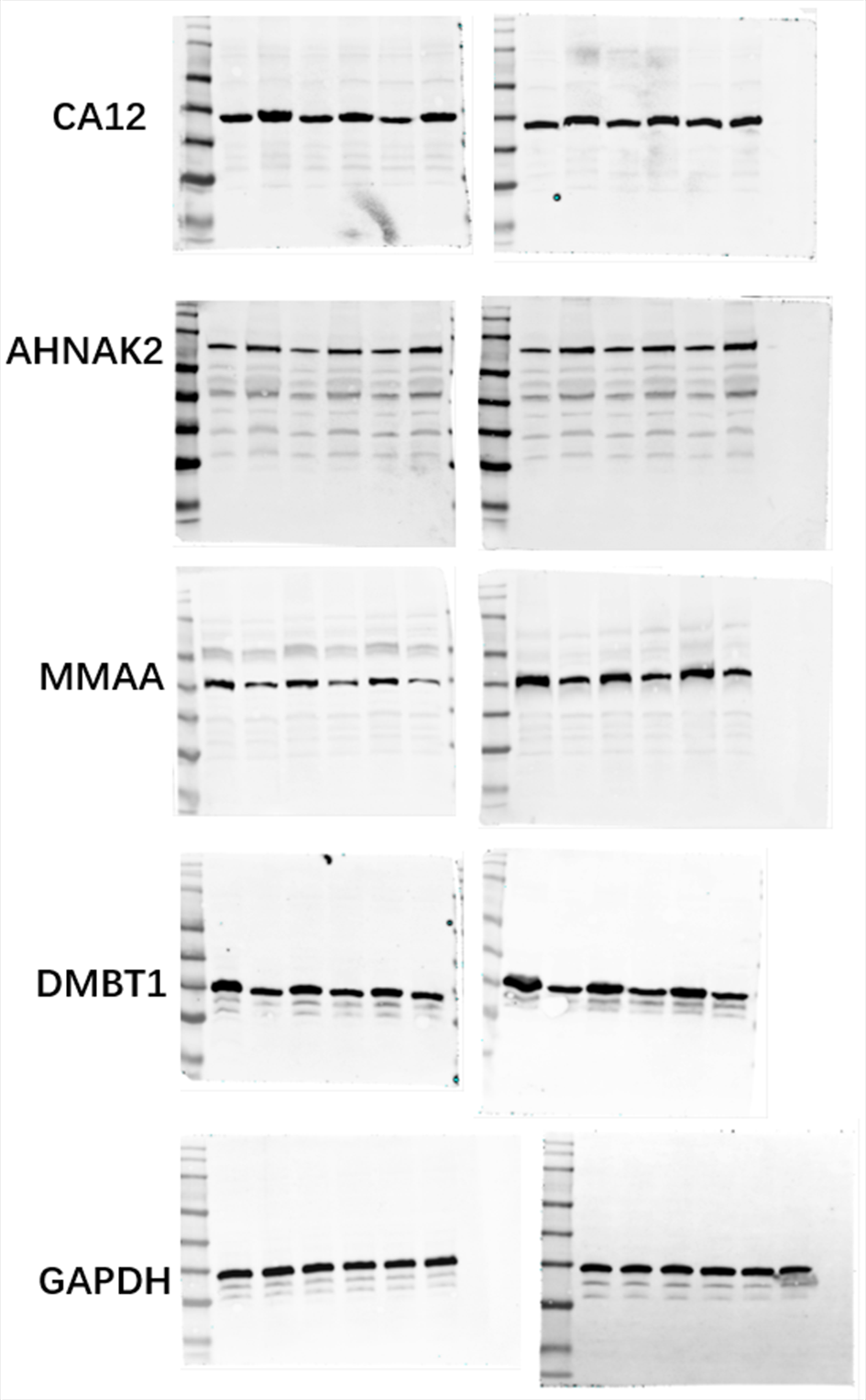

Supplement: Supplementary file 2 [file Image_2.tif]
